# Application of an In Vivo Hepatic Triacylglycerol Production Method in the Setting of a High-Fat Diet in Mice

**DOI:** 10.3390/nu9010016

**Published:** 2016-12-28

**Authors:** Kikumi D. Ono-Moore, Matthew Ferguson, Michael L. Blackburn, Hassan Issafras, Sean H. Adams

**Affiliations:** 1 Arkansas Children’s Nutrition Center, Little Rock, AR 72202, USA; kdonomoore@uams.edu (K.D.O.-M.); FergusonME@archildrens.org (M.F.); BlackburnMichaelL@uams.edu (M.L.B.); 2 Department of Pediatrics, University of Arkansas for Medical Sciences, Little Rock, AR 72202, USA; 3 XOMA Corporation, Berkeley, CA 94710, USA; hassan.issafras@merck.com

**Keywords:** triacylglycerols, non-alcoholic fatty liver disease, high fat, DIO, fatty acid synthase, poloxamer 407

## Abstract

High-fat (HF) diets typically promote diet-induced obesity (DIO) and metabolic dysfunction (i.e., insulin resistance, hypertriglyceridemia, and hepatic steatosis). Dysfunction of triacylglycerol (TAG) metabolism may contribute to the development of hepatic steatosis, via increased de novo lipogenesis or repackaging of circulating nonesterified fatty acids (NEFAs). Hepatic TAG production (HTP) rate can be assessed through injecting mice with nonionic detergents that inhibit tissue lipoprotein lipase. Potential confounding effects of detergent-based HTP tests (HTPTs) used in longitudinal studies—including the impact on food intake, energy balance, and weight gain—have not been reported. To examine this, male C57BL/6J mice were fed a 10% or 60% kcal diet. After 4 weeks, the mice underwent an HTPT via poloxamer 407 intraperitoneal injections (1000 mg/kg). Weight gain, energy intake, and postabsorptive TAG levels normalized 7–10 days post-HTPT. The post-HTPT recovery of body weight and energy intake suggest that, in metabolic phenotyping studies, any additional sample collection should occur at least 7–10 days after the HTPT to reduce confounding effects. Diet-specific effects on HTP were also observed: HF-fed mice had reduced HTP, plasma TAG, and NEFA levels compared to controls. In conclusion, the current study highlights the procedural and physiological complexities associated with studying lipid metabolism using a HTPT in the DIO mouse model.

## 1. Introduction

Diets high in saturated fat and simple sugars are associated with poor metabolic health in humans and, when coupled with obesity, increase the risk for development of chronic diseases and hyperlipidemia. The increased risk for cardiovascular disease associated with type 2 diabetes (T2D) is thought to be in part due to hyperlipidemia (both triacylglycerol (TAG) and cholesterol) [1]. The etiology of hypertriglyceridemia in the insulin-resistant state of prediabetes and early T2D involves, in part, hyperinsulinemia coupled to lipolysis in adipose tissue that promotes nonesterified fatty acid (NEFA) delivery to the liver [2,3]. Less clear is the impact of the typical diet on regulation of the pathways involved in liver lipid synthesis, packaging, and export.

Male C57BL/6J mice fed variations of a high-fat (HF), high-sucrose diet (i.e., 45%–60% kcal from fat) is a well-established diet-induced obesity (DIO) model used to study insulin resistance and other obesity-associated metabolic and inflammation sequelae (i.e., increased tumor necrosis factor (TNF)α and other proinflammatory cytokines, adipose inflammation, insulin resistance) [4,5,6,7]. For instance, this mouse model has been used to study obesity and insulin resistance-associated hepatic steatosis and nonalcoholic fatty liver disease (NAFLD) [8]. The degree by which liver TAG metabolism in the DIO setting is impacted by one or more of the following is an open question: increased de novo lipogenesis (DNL), lipogenesis from NEFA delivery to the liver, decreased β-oxidation [9], or reduced TAG export from the liver [3,7]. In theory, the contribution of each to modifying TAG homeostasis may differ, depending on specific components of the diet (i.e., amount of simple sugars or lipids), or degree of insulin resistance and insulin status that can affect adipose lipolysis and insulin action on liver lipogenic pathways.

To evaluate these questions as related to liver lipid metabolism, it is useful to consider in vivo assays of hepatic TAG production. To this end, we compared liver lipid synthesis and metabolism phenotypes in DIO mice fed a 60% fat diet and mice fed a low-fat (LF) control diet matched for simple sugar (sucrose) on percent of energy basis. The measurement of hepatic TAG production can be performed by injecting mice with a nonionic detergent that inhibits blood TAG hydrolysis by lipoprotein lipase (LPL) [10]. In fasted mice, the prevention of TAG clearance elicits a rise in circulating TAG, which is a surrogate for hepatic export of TAG (also known as hepatic TAG production). Several nonionic detergents have been used in the assessment of hepatic TAG production, such as Triton WR-1339 and poloxamer 407 (P-407) [10]. Triton WR-1339 has been shown to be hemolytic, while P-407 is reported to be less toxic [11]. In addition, P-407 can be administered through intraperitoneal (i.p.) injection instead of tail vein injections, making its delivery facile and reducing the stress to the animal. Despite the value of detergent-based hepatic TAG production tests (HTPT) to evaluate liver function in experimental models that examine effects of diet and obesity, methodological nuances should be considered for interpretation of metabolic phenotypes. Investigators using P-407, for instance, noted that fasting TAG levels return to pre-HTPT levels 96 h post-administration of P-407 [11,12]; however, this and other studies did not report if food intake and body weight gain return to pre-HTPT levels following detergent treatment [11,12]. Any transient or long-lasting changes in energy balance could confound additional important endpoints of a study. With these aspects in mind, the purpose of this study was to provide a more detailed picture of post-HTPT physiology and TAG patterns in C57BL/6J mice, in tandem with an application of the HTPT in the setting of HF and LF feeding to determine if DIO increases basal hepatic TAG production.

## 2. Materials and Methods

### 2.1. Study Design

**Figure 1:**
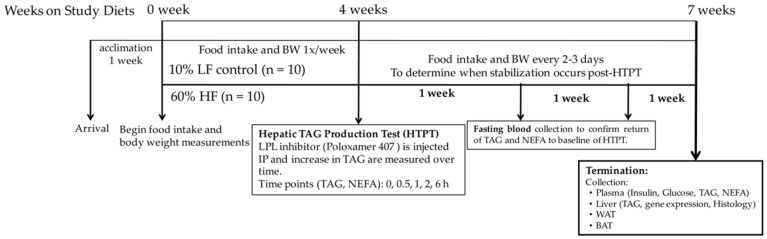
Study Schematic. BAT, brown adipose tissue; BW, body weight; HF, high fat; IP, intraperitoneal; LF, low fat; LPL, lipoprotein lipase; NEFA, nonesterified fatty acids; TAG, triacylglycerides; WAT, white adipose tissue.

All animal protocols were approved by the University of Arkansas for Medical Sciences Institutional Animal Care and Use Committee, according to Animal Welfare Act guidelines (17 July 2015, File# 3637). Figure 1 shows a schematic of the study design. Five-week old male C57BL/6J mice were purchased from Jackson Laboratory (Bar Harbor, ME, USA). Mice were single-housed, placed on a 12 h light and dark cycle, and had ad lib access to food and water. Mice were fed Teklad 8640 rodent chow (Envigo, Indianapolis, IN, USA) during the 1-week acclimation period. Starting at 6 weeks of age, mice were randomized to 60% kcal from fat diet (Research Diets D12492) or a 10% kcal from fat diet (Research Diets D12450J), matched for sucrose on percent of energy basis (Table 1) (Research Diets, Inc., New Brunswick, NJ, USA).

Food intake and body weight were measured once a week from the start of the study to the HTPT. These measures were conducted at approximately the same time each day (ca. 10:00–12:00). When there was a large amount of uneaten food in the cage, the bedding was sifted, and the uneaten food pieces were collected and measured. After the HTPT, intake and body weights were measured 3 days a week.

### 2.2. Hepatic Triacylglycerol Production Test (HTPT)

A P-407 (NF-grade) (Spectrum Chemicals, New Brunswick, NJ, USA, Cat# P1166, Lot# 2EC0122) solution (100 mg/mL) was prepared by mixing the appropriate amount of room-temperature P-407 with injection-grade saline (Baxter Healthcare Corp., Deerfield, IL, USA) under sterile conditions. The solution was placed in ice on a rocking platform to solubilize overnight. The P-407 solution was brought to room temperature before mice were injected.

After 4 weeks on their respective diets, mice were fasted starting at 05:00 for 5–6 h prior to undergoing a HTPT. For the HTPT, mice were injected (i.p.) with P-407 (1000 mg/kg) and blood was sampled via a tail vein nick into ethylenediaminetetraacetic acid (EDTA) Microvettes (Sarstedt, Nümbrecht, Germany) at baseline (0 min), 0.5, 1, 2, and 6 h [10]. Concentration of TAG was analyzed as described below. To monitor longer-term changes in TAG levels following the HTPT test day, postabsorptive (4–6 h fasted) blood draws were completed 1 and 2 weeks after the HTPT via a tail vein nick. At 3 weeks after the HTPT, mice were euthanized for tissue collection in the postabsorptive state. While sedated under isoflurane/O_2_, EDTA (Ambion, Life Technologies, Grand Island, NY, USA) plasma was collected via a cardiac puncture; mice then underwent a diaphragmectomy. EDTA plasma for measurement of postabsorptive insulin and glucose was collected immediately after centrifugation at 10,000× *g* for 2 min at 4 °C.

### 2.3. Blood Analyte Measurements

NEFAs and TAGs were measured using HR Series NEFA-HR(2) and L-Type TG M reagents, and the microtiter procedure supplied by the manufacturer (Wako Chemical USA, Richmond, VA, USA). Plasma insulin levels were determined using the Ultra Sensitive Mouse Insulin ELISA (Crystal Chem, Downers Grove, IL, USA). Plasma glucose levels were assessed with SynerMed colorimetric glucose assay (Synermed, Westfield, IN, USA). A BMG Labtech’s POLARstar Omega plate reader (Ortenberg, Germany) was used to obtain optical densities. The generation of standard curves and determination of unknown concentrations were done using Prism GraphPad v 6.0 for Mac OS X (GraphPad Software, La Jolla, CA, USA) for NEFA and TAG data, and MARS: Data Analysis Software (Ortenberg, Germany) for insulin and glucose.

### 2.4. Liver TAG Determination

Liver lipids were extracted using a modified Folch method [13]. Briefly, approximately 100 mg of liver was homogenized in 2:1 (*v:v*) chloroform:methanol (ACS grade, Fisher Scientific, Waltham, MA, USA) solution using a Precellys 24 bead beater (Bertin Instruments, Montigny-le-Bretonneux, France) (2 by 20 s at 5000 rpm with 2 min of rest on ice during the homogenization process). The addition of 0.7% saline (Baxter Healthcare Corp.) facilitated the separation of the organic phase, and samples were dehydrated overnight under nitrogen. The lipids were reconstituted in 1 mL of isopropanol (ACS grade, Fisher Scientific). The TAG content of the lipid extracts was measured using l-Type TG M reagents according to the microtiter procedure (Wako).

### 2.5. Total RNA Isolation and Gene Expression

Total RNA was isolated using the RNeasy Plus kit (Qiagen, Valencia, CA, USA) and cDNA was prepared using the iScript cDNA Synthesis Kit (Bio-Rad, Hercules, CA, USA). qRT-PCR utilized gene-specific primers (Table 2) and fast SYBR green master mix (Life Technologies, Grand Island, NY, USA). Relative standard curves were produced for each target by synthesizing cDNA from a pooled RNA sample (10 μL/mouse used in the pool). A five-point relative standard curve was produced for each target gene and for 18S ribosomal RNA, using Prism GraphPad, and cycle threshold (CT) values were used to determine relative abundance (arbitrary units). Gene expression was normalized to 18S ribosomal RNA (gene of interest arbitrary unit/18S arbitrary unit).

### 2.6. Immunoblotting

Approximately 100 mg of liver was homogenized in 500 µL of RIPA Buffer (Teknova, Hollister, CA, USA) with Halt protease and phosphatase inhibitors (Thermo Scientific, Waltham, MA, USA) and 1 mM phenylmethanesulfonyl fluoride (PMSF) (Sigma, St. Louis, MO, USA). Lysates were debrided at 15,000× g for 10 min at 4 °C. Protein was quantified using Thermo Scientific Pierce BCA Protein Assay (Waltham, MA, USA). Lysates were subjected to 6% SDS-PAGE (Tris-glycine) followed by transfer of the proteins to polyvinylidene difluoride membrane (Bio-Rad). Membranes were then blocked in PBS and 0.1% Tween-20 (*v/v*) (Fisher Scientific) containing 2% nonfat dry milk. The membranes were probed for 1 h or overnight with primary antibodies followed by incubation with horseradish peroxidase-conjugated secondary antibody (Southern Biotech, Birmingham, AL, USA) for 1 h. Protein was detected using ECL Clarity Western blot detection reagents (Bio-Rad) followed by imaging on an Amersham Imager 600 (GE Healthcare Bio-Sciences, Pittsburgh, PA, USA). Densitometry was determined using IQ-TL v8 software (GE Healthcare Bio-Sciences), and an amido black (Sigma, St. Louis, MO, USA) stained protein band of 150 kDa was used as the loading control. Normalization was completed by dividing the background-subtracted band of interest by the background-subtracted amido black band. Data are expressed relative to the LF control group. The following primary antibodies were used: fatty acid synthase (FASN) (sc-20140, Santa Cruz Biotechnology, Santa Cruz, CA, USA), acetyl-CoA carboxylase (ACC)1 (#3662, Cell Signaling Technology, Danvers, MA, USA), and microsomal TAG transfer protein (MTTP) (#612022, BD Transduction Laboratories, San Jose, CA, USA).

### 2.7. Histology

Frozen liver pieces were placed in OTC media, sectioned, and stained as described in Baumgardner et al. [14]. A blinded histology technician selected 10 random fields per mouse, and quantified the percentage of area stained by Oil Red O using MCID Imaging Software version 7.0 (MCID, Cambridge, UK) linked to an Olympus Bx50 microscope (Olympus, Pittsburgh, PA, USA). A representative image for each mouse is located in the Supplemental Material (Figure S1).

### 2.8. Statistical Analysis

Statistical analysis was completed using Prism GraphPad. When two means (LF vs. HF) were compared, a Student’s two-tailed *t*-test was used. Repeated measures two-way ANOVA was used to assess temporal differences in body weight and calorie intake. Bonferroni’s multiple comparisons test was used to assess differences across each time point. Linear regression was used to test whether the HTPT slopes were different. For correlations, Pearson’s correlation coefficient was determined. Outliers were removed based on GraphPad Prism’s robust regression and outlier (ROUT) removal test (*q* = 1%). All data are represented as mean ± SEM unless otherwise noted. *p* < 0.05 was considered significant.

## 3. Results

**Figure 2:**
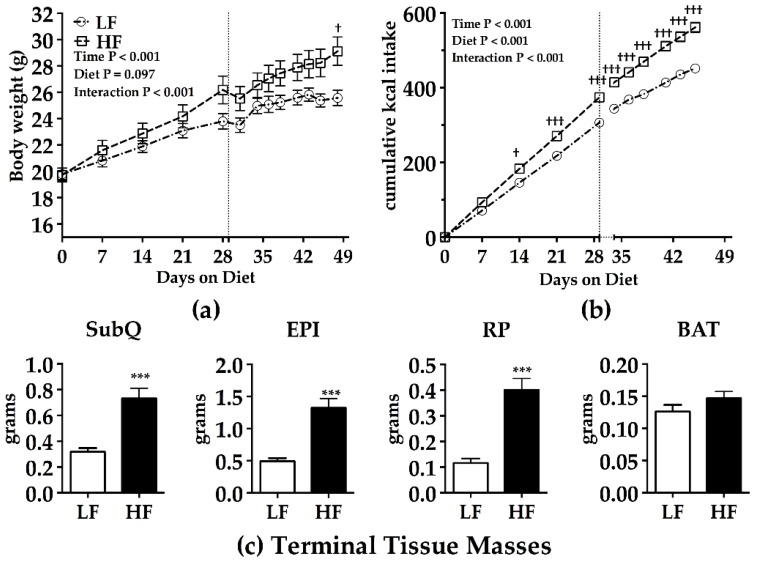
Body weight, terminal adiposity, and postabsorptive metabolic markers in male C57BL/6J mice fed a control or obesity-promoting diet for 7 weeks. (**a**) Body weight; and (**b**) food intake of male C57BL/6J mice fed a 60% kcal from fat diet (HF) or a 10% kcal from fat diet (LF) for 50 days (~7 weeks). The vertical line indicates when the hepatic triacylglycerol production test (HTPT) was completed. The break in the food intake *x*-axis indicates two days in which food was not collected; (**c**) Terminal tissue masses after 50 days on the respective diets. BAT, intrascapular brown adipose tissue; EPI, epididymal white adipose tissue, RP, retroperitoneal white adipose tissue; SubQ, subcutaneous fat; ^†^ *p* < 0.05, ^†††^ *p* < 0.001 Bonferroni’s multiple comparisons test; *** *p* < 0.001; Student’s two-tailed *t*-test. Mean ± SEM. LF *n* = 10; HF *n* = 10.

As anticipated, the HF-fed mice had increased body weight and cumulative energy intake compared to LF controls (Figure 2A,B). In turn, more fat and fewer total carbohydrates (total = simple plus complex carbohydrates) were consumed by the HF-fed mice than the LF controls (Table 3), but, notably, the HF-fed mice consumed 1.8 g more sucrose than the LF controls due to their higher overall recorded cumulative calorie intake (Table 3). Adiposity was significantly increased in the HF-fed mice compared to the controls (Figure 2C). HF-fed mice exhibited hyperinsulinemia; however, plasma glucose was modestly lower compared to LF fed mice after 7 weeks on the HF diet (Table 3). They also had lower plasma TAG and NEFA levels (Table 3).

**Figure 3:**
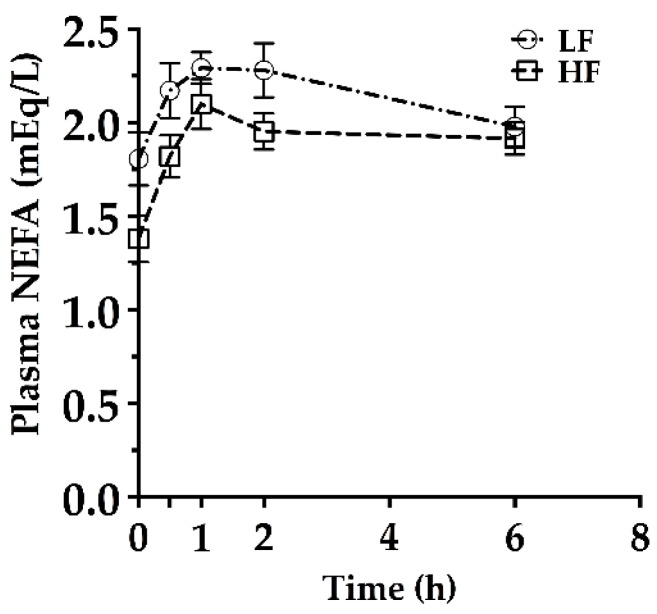
Plasma NEFA concentrations during the hepatic triacylglycerol production test (HTPT) in male C57BL/6J mice fed a low-fat (LF) or high-fat (HF) diet for 4 weeks. Mice were fasted 5–6 h prior to the HTPT (postabsorptive). Blood was sampled via a tail vein nick at 0 h (baseline), 0.5, 1, 2, and 6 h. One mouse was excluded from the NEFA data due to limited sample volume. Mean ± SEM. LF *n* = 9; HF *n* = 10.

**Figure 4:**
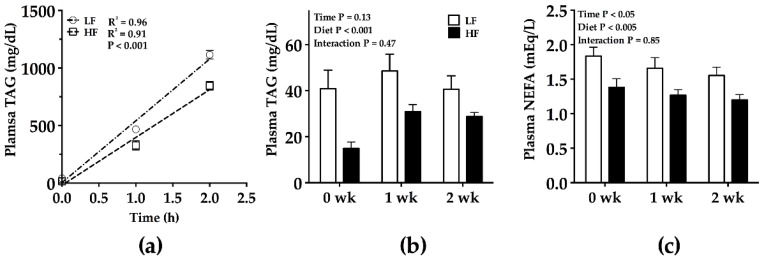
Hepatic triacylglycerol production test (HTPT) and plasma TAG and NEFA levels post-HTPT in male C57BL/6J mice fed a low fat or high fat diet. (**a**) HTPT in male C57BL/6J mice fed a low-fat (LF) or high-fat (HF) diet for 4 weeks; (**b**) postabsorptive plasma TAG; and (**c**) NEFA in the weeks following HTPT. Mice were fasted 5–6 h prior to the HTPT (postabsorptive). Blood was sampled via a tail vein nick. The slope of TAG production significantly differs between the low-fat- and high-fat-fed animals, as indicated in the key. One mouse fed the LF diet was excluded from the TAG data due to abnormally high levels of plasma TAG (3.5-fold higher than the animals) at 1 and 2 weeks post-HTPT. One mouse fed the LF diet was excluded from the NEFA data due to an error in collecting the sample. Data were analyzed using a two-way ANOVA (main effects, diet and time; interaction, diet by time). Mean ± SEM. LF *n* = 9; HF *n* = 10.

After approximately 4 weeks on the respective diets, an HTPT was conducted. The results of the HTPT indicated that at 30 min, i.p. P-407 did not adequately inhibit LPL systemically, since NEFA levels rose until 1 h, after which they essentially stabilized at least to the 2 h time point (Figure 3). The absolute TAG concentrations between LF- and HF-fed mice were equivalent at 6 h (2928 ± 149 mg/dL; 2884 ± 81 mg/dL, respectively), but the variability of TAG levels more than doubled from 2 h to 6 h (LF SEM, 33 to 149 mg/dL; HF SEM, 36 to 81 mg/dL). For these reasons, TAG concentration data from the 0, 1, and 2 h time points were used to calculate HTPT slopes (Figure 4A). Using this paradigm, HF-fed mice had lower hepatic TAG production compared to LF mice (Figure 4A), as well as lower postabsorptive (time 0 h) plasma TAG and NEFA concentrations (Figure 4B,C). There was a main effect of diet on postabsorptive TAG and NEFA levels 1 and 2 weeks after the HTPT (Figure 4B,C). There was no main effect of time on postabsorptive TAG levels, but postabsorptive NEFA levels were affected by time (time main effect *p* < 0.05). Body weight gain and energy intake were normalized 7–10 days post-HTPT (Figure 2A,B).

**Figure 5:**
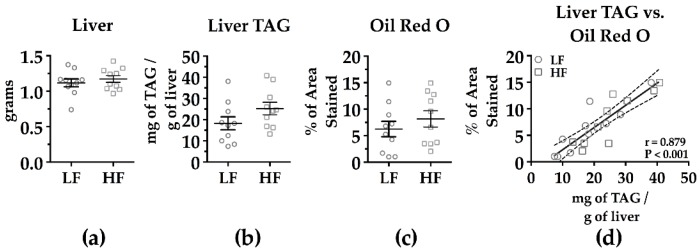
Liver mass (**a**); liver TAG content (**b**); percentage of area stained by Oil Red O (**c**); and the correlation between liver TAG and Oil Red O staining (**d**) in male C57BL/6J mice fed a low-fat (LF) or high-fat (HF) diet for 7 weeks prior to assessment. Mice were fasted 5–6 h prior to collection of the livers. See Figure S1 for representative Oil Red O images for each mouse. Pearson r is displayed. Mean ± SEM. LF *n* = 10; HF *n* = 10.

Differences in hepatic TAG production could be associated with differences in the liver TAG pool size, prompting an evaluation of liver TAG. There was no significant difference in the liver masses between the HF and LF groups (Figure 5A). Hepatic TAG levels (mg of TAG/g of liver) were higher in the HF-fed mice at 7 weeks on diet, but the difference did not reach statistical significance (Figure 5B). Hepatic TAG levels measured using Oil Red O staining also did not show a significant difference between the HF and LF groups (Figure 5C) (representative images Figure S1). As expected, there was a strong correlation (*r* = 0.879, *p* < 0.001) between both methods used to assess hepatic TAG levels (Figure 5D).

**Figure 6:**
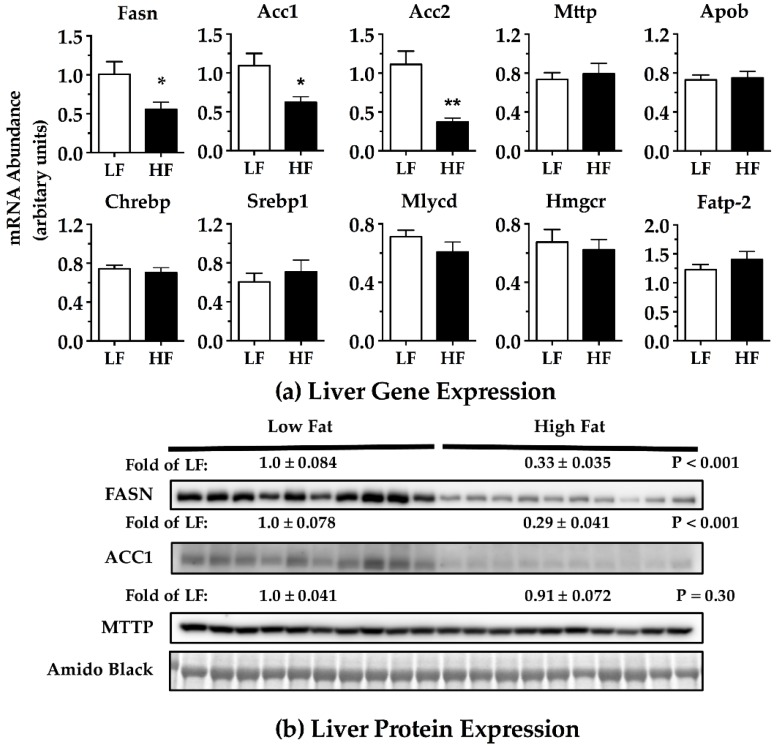
Liver fatty acid metabolism related gene (bar graphs) and protein (Western blots) expression in male C57BL/6J mice fed a low-fat (LF) or high-fat (HF) diet for 7 weeks prior to assessment. Gene expression was normalized to 18S RNA. Protein densitometry was normalized to an amido black stained protein band of approximately 150 kDa. Protein densitometry is displayed as fold of LF, mean ± SEM. Mice were fasted 5–6 h prior to collection of the livers. Abbreviations are listed in Table 2. * *p* < 0.05; ** *p* < 0.01. Student two-tailed *t*-test. LF *n* = 10; HF *n* = 10.

Differences in pathways involving DNL or lipid packaging and export could contribute to diet- or obesity-associated hepatic TAG production. As an initial evaluation of this possibility, a selection of fatty acid metabolism related genes in the liver were assessed via qRT-PCR and immunoblotting. Fatty acid synthase (Fasn) and acetyl-CoA carboxylase (Acc) 1 and 2 are key regulators of DNL and fatty acid oxidation, respectively. Fasn facilitates the elongation of acetyl-CoA units into a C16- or C18-fatty acid. Livers from HF-fed mice exhibited significantly reduced gene and protein expression of Fasn (Figure 6A,B). Acc1 and Acc2 catalyze the synthesis of malonyl-CoA [15]. Protein (Acc1) and gene expression of Acc1 and Acc2 were significantly reduced in HF-fed mice compared to LF-fed control mice (Figure 6A,B). Microsomal TAG transfer protein (Mttp) facilitates the transfer of TAG to immature ApoB lipoprotein [16,17]. Mttp expression at the mRNA and protein levels were not significantly different between HF- and LF-fed mice (Figure 6A,B). Furthermore, ApoB100 mRNA was not significantly different (Figure 6A). Fatty acid transport protein 2 (Fatp-2)—which facilitates the transfer of circulating NEFA into the liver—mRNA expression was not significantly different between HF- and LF-fed mice. There were no significant differences in the gene expression of other metabolic genes we assessed (carbohydrate-responsive element-binding protein (Chrebp), sterol regulatory element-binding protein 1 (Srebp1), malonyl-CoA decarboxylase (Mlycd), and 3-hydroxy-3-methylglutaryl-CoA reductase (Hmgcr)) (Figure 6A). Taken together with the liver TAG results, the data support the hypothesis that the reduced hepatic TAG production seen in the HF-fed mice compared to the LF-fed mice is due to a reduction in DNL and not due to impairment of TAG trafficking out of the liver.

## 4. Discussion

The liver is central to metabolism and acts as a homeostatic organ that helps maintain systemic energy homeostasis by varying the production rate of fuels such as fat and glucose. The hepatic production rate of TAG can be influenced by dietary sources of carbohydrate (i.e., stimulated by high intakes of simple sugars such as sucrose [18,19]), and disease states (type 2 diabetes, obesity, and insulin resistance) [1]. The hepatic production of TAG is dependent on two sources of NEFA: endogenously produced via DNL and uptake of circulating NEFA that are repackaged into TAG. The liver will increase DNL to convert dietary carbohydrates into fatty acids for export and transport to adipose tissue storage under carbohydrate-rich conditions that are typically accompanied by high insulin (i.e., the postprandial state). Subsequently, the rate of DNL may be reduced under lipid-rich conditions (HF diets) in which insulin concentration can be more modest than carbohydrate-rich conditions [20].

T2D and insulin resistance are associated with increased circulating TAG and NEFA levels and hepatic steatosis [21,22]. The mechanisms behind hepatic steatosis resulting from insulin resistance may involve one or more of the following: high insulin levels upregulating hepatic genes that promote DNL and very low-density lipoprotein (VLDL) production, increased delivery of diet-derived NEFA to liver, and/or reduced export of TAG [3]. Insulin resistance can also increase the net breakdown of stored TAG in adipose tissue, increasing the circulating NEFA levels and providing another source of fatty acids for packaging and export from the liver [23]. Characterizing animal models and methods to determine net liver TAG production is important to understand mechanisms underlying diet- and obesity-associated metabolic shifts, and has relevance to evaluation of in vivo efficacy of nutritional and other interventions targeted to improve liver health or prevent disease. Models that enable hepatic TAG production evaluations over time, or that allow for longitudinal metabolic physiology measures following TAG production assays, will be especially valuable.

Herein, we provide a first-ever assessment of temporal energy balance and body weight trajectories following measurement of in vivo hepatic export of TAG through the injection of a nonionic detergent. The nonionic detergent used herein, P-407, inhibits a variety of lipases including pancreatic lipase [24] and hepatic lipase, in addition to LPL [25]. P-407 inhibition of LPL prevents circulating TAG from being broken down and absorbed by adipose for storage [10]. The HTPT has been widely used to assay hepatic TAG production or more accurately the export of hepatic TAG in rodent models [7,10,11,12]. This study was designed in part to assess a reasonable time frame post-HTPT in mice to minimize the confounding effects of test-associated stress on gross metabolic parameters such as body weight and food intake. Our results indicate that these parameters are normalized by ≈7–10 days post-HTPT in both HF-fed DIO mice and LF diet-fed male C57BL/6J mice. Postabsorptive plasma TAG levels also normalized within about a week. Time did have a limited impact on NEFA levels post-HTPT; whether time effect reflects a residual effect of the P-407 detergent on LPL activity or an age-associated pattern cannot be fully ascertained using the study design herein, and this question will await future studies that focus on this aspect. Overall, our data suggest that to minimize artefacts from the HTPT on metabolic endpoints, at least 7–10 days post-HTPT is required in mice.

In addition to evaluating residual effects of HTPT on metabolism, we also evaluated hepatic TAG production in HF-fed mice compared to LF-fed mice. At first blush, it would be expected that a HF diet and obesity would increase TAG export and circulating levels of TAG and NEFAs. In fact, mice fed an HF diet for 4 weeks had reduced hepatic TAGs production and lower postabsorptive circulating TAG and NEFA levels, despite hyperinsulinemia (measured at 7 weeks on diet). This outcome was not anticipated, but in retrospect is likely explained by considering several possible mechanisms. First, the reduction in hepatic TAG production in HF-fed mice might be attributed to reduced DNL pathways in response to the HF environment, as these mice had reduced liver expression of DNL-critical genes *Fasn* and *Acc1* compared to LF-fed controls. High-fat-fed rats and mice reportedly have reduced expression of lipogenic genes, as well [26,27]. These genes are especially responsive to insulin and high-sucrose or high-fructose diets in rodents [28,29,30]. Fructose is thought to contribute to DNL in part due to bypassing the regulatory step in glycolysis (phosphofructokinase) [31], and the *Fasn* and *Acc1* genes contain carbohydrate response elements [32]. Importantly, dietary sucrose as a percent of kcal was matched between the two diets in the current study, to remove the confounding effect of dietary glucose/fructose on liver DNL. The HF-fed mice, due to their overall higher energy intake, consumed on average 1.8 g more sucrose than the LF controls; yet, overall, they consumed less total carbohydrate (51 g) during the study. Thus, the higher TAG production in mice fed the LF diet relative to mice fed a HF diet could be due to an increase in total dietary carbohydrate in LF mice, coupled to relatively higher *Fasn* and *Acc1* gene expression. Other studies that directly measured liver DNL in mice fed high-fat diets showed reduced DNL, with increased elongation and triglyceride synthesis contributing to hepatic steatosis [33,34]. A second possibility is that reductions in hepatic TAG production and reduced circulating TAG in DIO mice stem from impaired export of TAG, which in turn could promote liver steatosis. However, this seems unlikely in the current study since expression of *Mttp* and *ApoB100*, key factors that regulate the export and production of VLDL [16,17], were not changed in the liver of mice fed an HF diet compared to controls. A recent study showed that mice fed a HF diet for 16 weeks exhibited reduced hepatic TAG production with an accompanying significant increase in hepatic steatosis, and reduced *ApoB* expression, suggesting that the decreased hepatic TAG production is due to a reduction in export [7]. In our study, gross liver TAG accumulation—as measured by biochemical assay or Oil Red O staining—was not statistically significantly different, in contrast to many studies that report increased liver TAG accumulation as early as 3 days to 1 week after the induction of high-fat feeding [35,36,37]. Differences between those studies and ours with respect to liver TAG accumulation might be due to differences in the type of diet used (types of carbohydrates and sources of fat). It is also important to note that the amount of time the diets were fed to the mice in the current study was shorter than the 10+ weeks used in many DIO studies [4,5,7,38]. The shorter time frame may not have led to gross insulin resistance that would typically promote accelerated lipolysis or loss of blood-sugar control (postabsorptive plasma glucose at the time of study termination was not higher in DIO mice: 133 vs. 121 mg/dL). Without gross insulin resistance, delivery of dietary fat to adipose tissue and the postprandial inhibition of adipocyte lipolysis may not have been perturbed, thereby preventing substrate-driven liver lipid accumulation in the HF diet condition [26]. In addition, Fatp-2, a protein that is involved in NEFA uptake by the liver, was not increased in DIO mice, suggesting the DIO livers had not compensated for increase substrate delivery at 7 weeks.

There are many studies that report increased DNL in patients with NAFLD [23,39,40]. On the surface, the data on expression of DNL-relevant genes and proteins, and the HTPT outcomes presented in this study and others [7,26,27], are contradictory. Many studies that report increased DNL or changes in hepatic TAG metabolism in patients with NAFLD use a comparison group that is well matched for obesity, sex, age, and other factors [39,40], or simply quantify the contribution of DNL to hepatic steatosis [23]. However, when diet and metabolic status are considered, differences in DNL become more nuanced. Schwarz et al. [41] observed that obese hyperinsulinemic subjects had increased DNL relative to obese and lean normoinsulinemic subjects under a low-carbohydrate diet, but that under a high-carbohydrate diet, lean normoinsulinemic subjects had an even greater increase in DNL. In our study and many other rodent studies, the control group is fed substantially more carbohydrate than the HF group (70% vs 20% kcal from carbohydrates in this study), suggesting that high-carbohydrate consumption might be more influential than obesity or hyperinsulinemia on DNL. The observation of Schwarz et al. [41] that a high carbohydrate diet increases DNL is well known and reviewed in [42], and holds true under eucaloric conditions [43]. In addition, low-carbohydrate diets (especially simple carbohydrates) can reduce circulating TAG compared to high-carbohydrate low-fat diets [44]. Taken together, our study does not contradict human NAFLD studies that show increased DNL, but is rather a reminder that the impact of gross macronutrient consumption should not be ignored when designing and interpreting DIO studies.

This study provides insight into the differences in export of hepatic TAG and DNL under LF and HF conditions; however, it has raised several questions that need to be addressed in future studies: (1) How quickly does the diet-associated effect on liver TAG production occur? In the current study, HF-fed animals were more obese and insulin-resistant when TAG production was measured, making it more difficult to attribute effects solely to diet alone; (2) Does the physiological reduction in HTP under high-fat/low-carbohydrate diet predispose the liver to impaired TAG export under high lipolysis conditions caused by worsened insulin resistance or frank diabetes? (3) What is the basis for interindividual variability in liver TAG accumulation, and would this contribute to differences in steatosis and nonalcoholic steatohepatitis (NASH)-like outcomes observed in human and animal studies?

## 5. Conclusions

In summary, our results are consistent with the hypothesis that reduced hepatic TAG production and lower TAG and NEFA levels in 7-week HF-fed DIO mice can be attributed to lower DNL compared to LF mice, coupled to relatively reduced lipolysis in the face of hyperinsulinemia. The hepatic TAG production test highlights the complexity and nuance associated with studying lipid metabolism in a DIO mouse model, viz. the importance of specific dietary components, time frame of diet intervention, and tissue-specific regulation of lipid metabolism in driving liver TAG production. The results also indicate that care should be taken to consider post-HTPT effects on body weight and energy intake if this test is being applied during longitudinal metabolic phenotyping experiments in mice.

## Figures and Tables

**Table 1:** Diet composition ^1^.

	Diet (Product Code)	
	Control (D12450J)	High Fat (D12492)
**Ingredients (g/kg)**		
Casein, 30-Mesh	190	258
l-Cysteine	2.84	3.88
Corn Starch	480	0.00
Maltodextrin 10	118	162
Sucrose	65.2	88.9
Cellulose, BW200	47.4	64.6
Soybean oil	23.7	32.3
Lard	19.0	317
Mineral Mix S10026	9.48	12.9
Dicalcium Phosphate	12.3	16.8
Calcium Carbonate	5.21	7.11
Potassium Citrate, 1 H_2_O	15.6	21.3
Vitamin Mix V10001	9.48	12.9
Choline Bitartrate	1.90	2.58
FD&C Yellow Dye #5	0.04	0.00
FD&C Red Dye #40	0.00	0.00
RD&C Blue Dye #1	0.01	0.07
**Macronutrients (% by weight)**		
Protein	19.2%	26.0%
Total Carbohydrate	67.3%	26.0%
Sucrose	6.52%	8.89%
Fat	4.3%	35.0%
**Macronutrients (% kcal)**		
Protein	20%	20%
Total Carbohydrate	70%	20%
Sucrose	6.8%	6.8%
Fat	10%	60%
kcal/g	3.85	5.24

^1^ Diets formulated and produced by Research Diets, Inc. (New Brunswick, NJ, USA).

**Table 2:** Forward and reverse primers used for qRT-PCR ^1^.

Gene	Gene Name	Forward	Reverse
18S	18S ribosomal RNA	GAGGCCCTGTAATTGGAATGAG	CGCTATTGGAGCTGGAATTACC
ApoB	Apolipoprotein B	ATACCACGTTTGCAAGCAGAAGCC	TGTTGAGCCGTAAGCTGTAGCAGA
Acc1	Acetyl-CoA Carboxylase 1/alpha	TAACAGAATCGACACTGGCTGGCT	ATGCTGTTCCTCAGGCTCACATCT
Acc2/Acacb	Acetyl-CoA Carboxylase 1/beta	AGTCTTCCGTGCCTTTGTAC	TTCTGCAAACTCATCCCTCG
ChREBP/Mlxpl	Carbohydrate-responsive element-binding protein	CATCTCCAGCCTCGTCTTC	CTTGGTCTTAGGGTCTTCAGG
Fapt-2	Fatty Acid Transport Protein 2	AGTACATCGGTGAACTGCTTCGGT	TGCCTTCAGTGGAAGCGTAGAACT
Fasn	Fatty Acid Synthase	TGACCTCGTGATGAACGTGTAC	GGGTGAGGACGTTTACAAAGG
Hmgcr	3-Hydroxy-3-Methylglutaryl-CoA Reductase	GCCCTCAGTTCAAATTCACAG	TTCCACAAGAGCGTCAAGAG
Mlycd	Malonyl-CoA Decarboxylase	CTCGGGACCTTCCTCATAAAG	CTCCTTCCCCTGCACATTC
Mttp	Microsomal Triglyceride Transfer Protein	TTCCCAGTAGGTTGGCTTTC	CACCTGGTTCACCCTGTTTA
Srebp1	Sterol regulatory element-binding protein 1	GGCTATTCCGTGAACATCTCCTA	ATCCAAGGGCATCTGAGAACTC

^1^ Purchased from Integrated DNA Technologies, Coralville, IA, USA.

**Table 3:** Cumulative macronutrient intake and postabsorptive plasma metabolic markers from male C57BL/6J mice fed a high-fat or low-fat diet for 7 weeks.

	Low Fat	High Fat	*p* Values
Cumulative Macronutrient Intake			
Total Carbohydrate (g)	79.1 ± 1.1	28.1 ± 0.7	<0.001
Sucrose (g)	7.7 ± 0.1	9.5 ± 0.2	<0.001
Fat (g)	5.0 ± 0.1	37.4 ± 0.9	<0.001
Protein (g)	22.6 ± 0.3	28.1 ± 0.7	<0.001
Plasma Metabolic Markers			
Insulin (pg/mL)	376 ± 24	636 ± 38	<0.001
TAG (mg/dL)	80.2 ± 8.00	47.4 ± 3.86	0.003
NEFA (mEq/dL)	0.644 ± 0.037	0.441 ± 0.027	<0.001
Glucose (mg/dL)	133 ± 2.38	121 ± 1.55	<0.001

Student’s two-tailed *t*-test. Mean ± SEM. LF *n* = 10; HF *n* = 9–10.

## Supplementary Materials

The following are available online at http://www.mdpi.com/2072-6643/9/1/016/s1, Figure S1: Oil Red O-Stained Liver Sections of male C57BL/6J mice fed low-fat or high-fat diets for 7 weeks (animal identifiers below images; diet information can be found in the Methods).
