# Supplementary material for: Application of an In Vivo Hepatic Triacylglycerol Production Method in the Setting of a High-Fat Diet in Mice
Source: Nutrients. 2016 Dec 28;9(1):16. doi: 10.3390/nu9010016 (PMC5295060; doi:10.3390/nu9010016)
Supplement: Supplementary file 1 [file nutrients-09-00016-s001.docx]

Supplementary Materials: Application of an In Vivo Hepatic Triacylglycerol Production Method in the Setting of a High-Fat Diet in Mice

Kikumi D. Ono-Moore, Matthew Ferguson, Michael L. Blackburn, Hassan Issafras and
Sean H. Adams


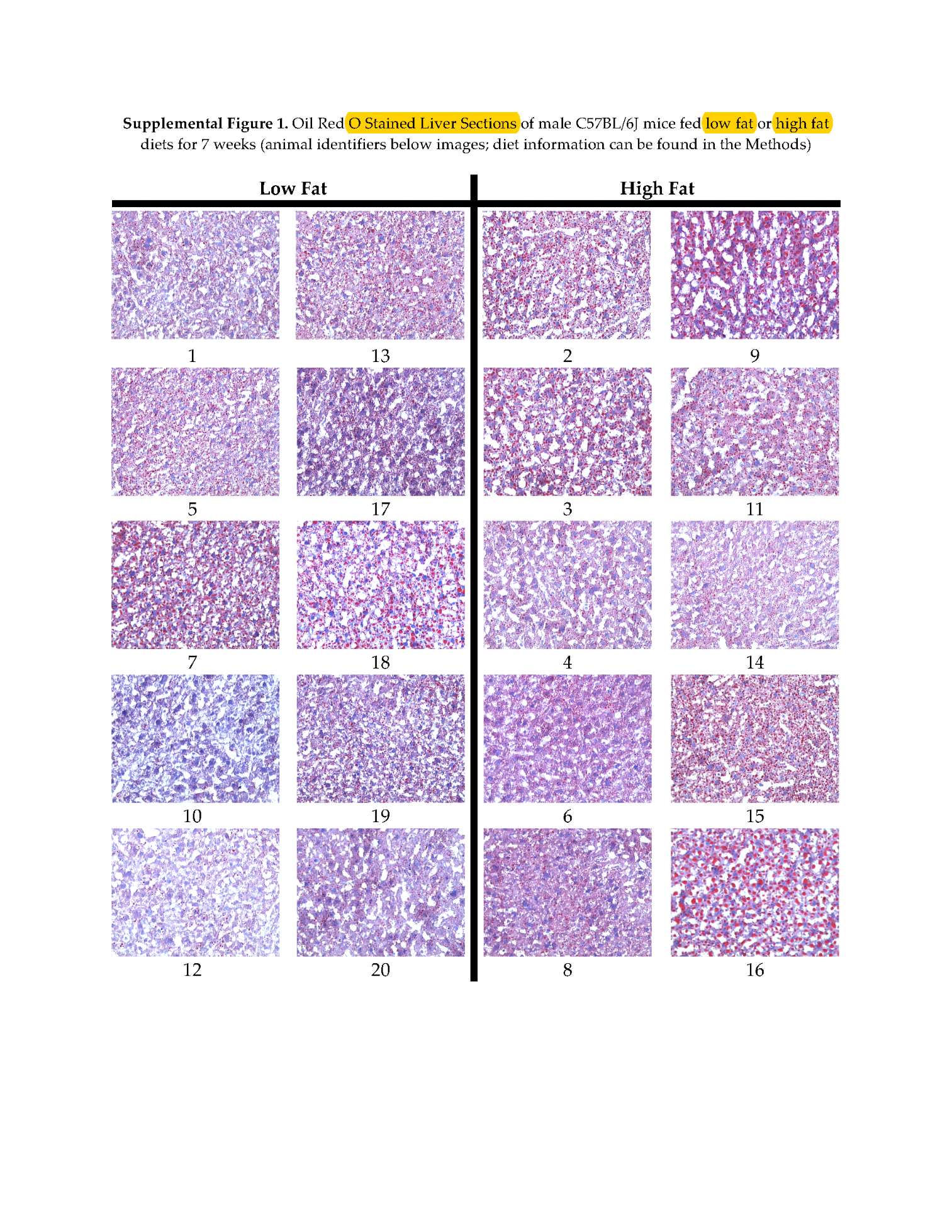


**Figure S1.** Oil Red O-Stained Liver Sections of male C57BL/6J mice fed low-fat or high-fat diets for 7 weeks (animal identifiers below images; diet information can be found in the Methods).
